# LocoBox: Modular Hardware and Open-Source Software for Circadian Entrainment and Behavioral Monitoring in Home Cages

**DOI:** 10.3390/s23239469

**Published:** 2023-11-28

**Authors:** Vuong Hung Truong, Jihwan Myung

**Affiliations:** 1 Graduate Institute of Mind, Brain and Consciousness (GIMBC), Taipei Medical University, New Taipei City 235, Taiwan; 2 Brain and Consciousness Research Centre (BCRC), TMU-Shuang Ho Hospital, New Taipei City 235, Taiwan; 3 Graduate Institute of Medical Sciences (GIMS), Taipei Medical University, Taipei 110, Taiwan

**Keywords:** circadian rhythms, entrainment, activity monitoring, T-cycle

## Abstract

Day–night locomotor activities are the most readily observed outputs of the circadian (~24-h period) clock in many animals. Temporal patterns of the light–dark schedule serve as input to the clock. While circadian activity patterns under various lighting conditions have been observed and documented, the full extent of circadian locomotor activities by genotype and entrainment remains uncharacterized. To facilitate large-scale, parallel cataloging of circadian input–output patterns, we created the LocoBox, an easy-to-construct and easy-to-operate system that can control environmental light with flexible entrainment scenarios combined with the T-cycle and measure locomotor activities in individual home cages. The LocoBox is made using economical, common components, and normal breeding cages can be used for long-term recording. We provide details of the components and blueprints, along with software programs for Arduino and a Python-based graphical user interface (GUI), so that the system can be easily replicated in other laboratories.

## 1. Introduction

From the early days of circadian biology, it has been a tradition to rely on naked-eye observations and to use readily available materials for experiments. Colin Pittendrigh described his first temperature compensation experiment in an outhouse and in a pressure cooker immersed in cold stream water, where he observed almost temperature-independent eclosion rhythms of fruit flies [1]. This experiment demonstrated one of the fundamental properties of circadian rhythms, yet it did not require a large budget. Many other properties and structures of internal circadian clocks have subsequently been inferred from locomotor activity plots (actograms). We now know that these inferences, made without knowledge of the underlying neural structures, were surprisingly accurate: for example, the central circadian clock operates as a dual-clock system that enables tracking of the time of the year [2].

Revolutions in circadian biology began with forward genetic screens in fruit flies, necessitating large-scale behavioral screening [3]. The tradition of using common materials continued in this screening as well, and when Yoshiki Hotta made the first locomotor activity monitor for drosophila, it was out of components from a local Radio Shack [4]. We now know that the fruit fly circadian clock system is not a simple 24-h clock, and, just as Pittendrigh predicted, dual clocks trace morning and evening to encode day-length-dependent changes in time [5]. Similar systems have been developed for mammalian forward genetics, specifically for the inbred mouse strain C57BL/6J [6]. This strain is known for its robust circadian free-running behavior under constant darkness, which shows a mean period of 23.7 h with a standard deviation of a mere 10 min [7]. Like the Drosophila Activity Monitoring (DAM) system (TriKinetics, Waltham, MA, USA) [8], Schenck et al. devised tunneled rodent housing in 1978, where movement is counted whenever a rat crosses an infrared beam [9]. In the 1960s, Kavanau developed several innovative automated recording devices for specific behaviors, such as drinking and feeding [10]. However, the sophistication of these devices and the complexity of their data were not suitable for the large-scale screening required by circadian phenotyping, especially considering that a single circadian phenotyping session can take about a month. Circadian phenotyping primarily focuses on characterizing the free-running period under constant darkness. Wheel-running measurement, due to its ease of parallelization, has become a popular method for forward genetics screening [11]. In this pipeline, the collected locomotor activity data are then processed to determine circadian periodicity using the chi-square periodogram in the ClockLab system (Actimetrics, Wilmette, IL, USA). The specialized wheel-running system is costly, and since it is in direct contact with animals, it requires frequent cleaning and maintenance. There is also a question of circadian periodicity change under wheel-running exercise [12]. General activity monitoring using the passive infrared (PIR) sensor was developed for situations where wheel-running is not feasible. It has been used for activity measurements under threat [13] and elderly mice nearing natural death [14]. The PIR sensor does not measure specific activities but rather general changes in the field, and it does not require frequent maintenance. The system has also been used for circadian phenotyping, but it remains costly (‘under $25,000 to monitor 24 animals at a time’ as of 2016) [15].

On the other hand, there is a need for detailed manipulation of the lighting condition in circadian phenotyping. Light, also called Zeitgeber (time-giver), is the primary entrainer of the circadian rhythm, and its strength and duration can modulate circadian activity patterns in different seasons and latitudes on Earth [16]. In mice, the clock neurons in the dorsal (shell) and the ventral (core) regions of the suprachiasmatic nucleus (SCN) exhibit different phase relations to encode seasonal time [17]. These internal network dynamics can create various patterns of circadian locomotor activities, and one example is jet lag. Circadian biologists have observed these patterns using standard protocols of light–dark schedules, which include constant dark (DD), constant light (LL), photoperiodic entrainment under variable ratios of light and dark durations (short day-length or long day-length), as well as short light pulses and jet lag protocols. However, these light schedules are only a small part of many other possible light–dark scenarios.

Importantly, by introducing independent internal and external timekeeping processes in light scheduling, our LocoBox software (BTLocoBox_0003_v3) provides an unparalleled flexibility in setting up the entrainment scheme. All conventional entrainments, including jet lag and long/short photoperiods, are achievable on the adaptable T-cycle platform. For instance, jet lag can be simulated in a T = 25 h cycle, approximating the transmeridian travel conditions on Mars. This unique feature allows for a variety of entrainment scenarios in combination with the T-cycle. To the best of our knowledge, no other tool currently offers such capabilities.

Characterizing circadian behaviors under many different light schedules within a single laboratory is challenging. This is because it necessitates meticulous examination of small alterations of patterns, such as activity bouts, and since one cycle is about 24 h, the recording duration of a single experiment can span months. To collaboratively explore the expansive parameter space of entrainment, we need an economical, open-source solution for a standardized small dark room, much like the outhouse used by Pittendrigh, but of a smaller size that houses a standard breeding cage. The standardized open-source setup we propose could enable researchers to systematically investigate circadian behaviors across a wide range of light conditions. Such a setup could expand opportunities for collecting circadian data from mice with various genetic backgrounds and environmental conditions while they remain in their breeding cages.

## 2. Materials and Methods

***Animals***: To assess the performance of LocoBox, individual wild-type C57BL/6J mice were housed in separate home cages, each placed inside a LocoBox. This mouse strain is the most commonly used in biomedical research and genetics studies [6,7,15,18]. Before initiating the experiments, the animals were maintained in a standard breeding room under 12:12 light–dark (LD) conditions (lights on at 7:00 and off at 19:00) and were subsequently transferred to the LocoBox in their respective cages. The animal study was conducted following protocols approved by the Institutional Animal Care and Use Committee (IACUC) of Taipei Medical University (LAC-2017-0469 and LAC2022-0477).

***LocoBox Housing***: Matte black Plexiglas sheets, 3 mm thick, were laser cut and assembled into a light-sealed box intended to house a breeding home cage for up to five mice. The front door was fitted with a light-shielding layer, while the back panel integrated a design that prevented external light from entering but ensured ample airflow (see Hardware Design).

***Electronic Components***: Each LocoBox was equipped with a silent DC brushless fan (Sunon Fans, Taiwan) in the back panel opening to facilitate outflow ventilation. Additionally, an LED (natural white, CCT 5000K; Cree LED, Durham, NC, USA) mounted on a heat sink (Wakefield-Vette, Nashua, NH, USA) was paired with a diffuser lens (Khatod, Milan, Italy) and was centrally positioned on the box’s ceiling. This design ensured uniform light distribution and the inclusion of the heat sink prolonged LED functionality during both light–dark cycles and constant light (LL) conditions. The LED could be turned on or off using a relay switch (reed relay for silent operation; Seeed Studio, Shenzhen, China), and a passive infrared sensor (PIR) on the ceiling monitored locomotor activities (Seeed Studio, Shenzhen, China). All these components were connected to the Arduino Mega 2560 (Arduino, Ivrea, Italy), which served as the interface between the electrical components and the data-logging and control computer. The real-time clock (RTC) DS3231 (Adafruit Industries, New York, NY, USA) was added to the Arduino for temperature-compensated precision timing. All data and power connections were made using JIT PH 4-pin connectors (Seeed Studio, Shenzhen, China). A standard 2N3904 NPN transistor (Onsemi, Scottsdale, AZ, USA) was used to create a current source for the LEDs. These components can be put together for about $100–150 per LocoBox (Table 1).

***Software Versions and Configurations Tested***: There are two parts to the software that operates the LocoBox. The first is the Arduino software (IDE version 1.8.7), which autonomously controls the sensors and relay switch outputs with its own internal clock using RTCLib for the DS3231 RTC (Table 1). The second is the Python software (version 3.7.0) which sets the lighting schedule and logs data on the computer through a GUI interface; it connects to the Arduino via serial communication through pySerial 3.4. Tkinter Library creates the graphical user interface (GUI) (already installed with Python 3.7.0 for Windows users). The LocoBox Python interface program was compiled (using PyInstaller 5.2) and tested with operating systems Windows 7, Windows 10, Ubuntu 16.04 LTS, and macOS 10.14. Coding was performed on Visual Studio Code (version 1.28.1) with Arduino and Python extensions. All software can be downloaded and further developed from GitHub at https://github.com/JihwanMyung/LocoBox.

### 2.1. Hardware Design

**Figure 1:**
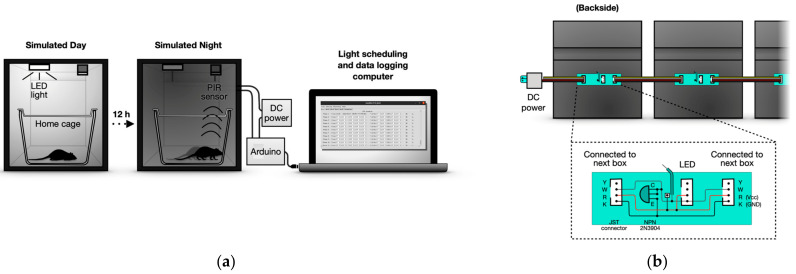
**Schematics of the LocoBox operation**. (**a**) A single LocoBox, operated by an Arduino, controls light cycles to mimic equatorial photoperiods. Diurnal activities, captured by the PIR sensor, are logged for later actogram creation. (**b**) Parallel LocoBoxes power the LED through short JST cables to neighboring units, eliminating the need for multiple DC source connections. A constant current unit for LEDs supports these connections. The diagram explains circuit details (inset).

An individual dark box made of black plexiglass serves as the LocoBox housing for a home cage. The box is completely light-sealed and is equipped with electronic components, such as a ventilating fan (which pumps air out of the box), a PIR sensor, and an LED with a heat sink and light diffuser (Figure 1). The plates used to construct the boxes are 3 mm thick, dark, and completely opaque. The interior is black and non-reflective.

**Figure 2:**
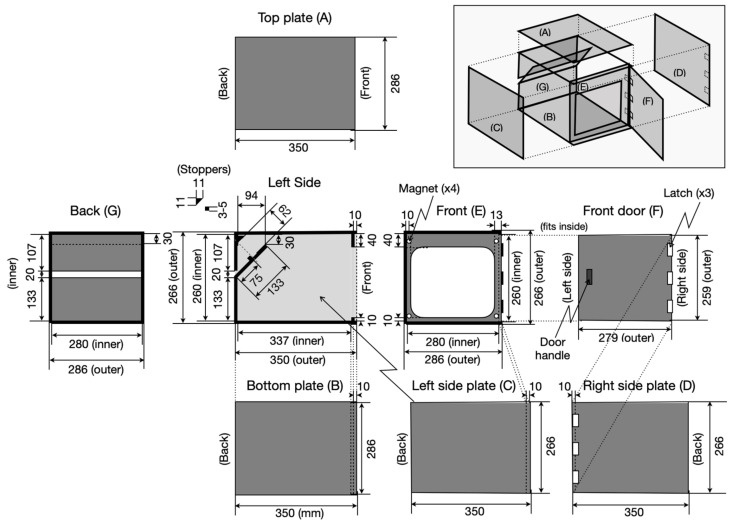
**Blueprint of LocoBox hardware**. The LocoBox is essentially a light-sealed box that allows airflow and provides entry points for various cables. Its slit can accommodate multiple cable sizes, and the box is spacious enough to fit a home cage with additional room for side devices. It consists of seven plates (A–G), constructed as shown in the inset illustration. The door is sealed with four small neodymium magnets. To ensure a light seal, a light barrier is placed between the door and the inside of the LocoBox. All units are in millimeters (mm).

Two features distinguish the LocoBox from conventional light-sealed boxes. The first is its relatively large ventilation slit on the back plate of the box, which isolates the box from the external environment through a double barrier structure. Additionally, the front door employs a similar double-barrier structure to prevent light leakage into the box. The LocoBox design is fully standardized and can be constructed entirely from the provided blueprint (Figure 2). The standardization of box dimensions is important because it ensures a consistent environment for the animals. The ventilation slit is designed to minimize internal humidity accumulation, even in the event of a ventilating fan malfunction. It also accommodates multiple cables, allowing for various measuring devices to be installed inside the box.

Another feature is the use of an individual current source that can be connected to adjacent current sources for easy parallelization, meaning only one of the boxes needs to be connected directly to the DC power source (Figure 1a). The current source design allows for daisy-chaining of power to LEDs and fans by connecting neighboring LocoBoxes, reducing the need for long extended cables to a common power source. In our current setup, a single Arduino manages five LocoBoxes simultaneously through digital input and output ports. This means data cables still need a central connection to the Arduino, leaving some cabling complexities unresolved. Despite remaining challenges, the replicable design and the ease of parallelization make the LocoBox a versatile tool for standardized long-term locomotor activity monitoring.

### 2.2. Software Design

The Locobox operates on a dual software architecture: one for the Arduino and another for the computer interface. The code that runs the Arduino microprocessor receives digital input from a PIR sensor and sends out digital output to a relay switch that controls an LED based on a timed schedule loop. An RTC, connected through the I2C bus, maintains the current time and serves as a timing reference for this schedule. The present LocoBox configuration employs one piece of Arduino software to simultaneously manage five entrainment boxes, each with independent lighting schedules. On the computer side, the Python interface establishes and sends the schedule parameters to the Arduino at the start of its operation, before the loop begins. During this initialization phase, the RTC also synchronizes with the computer’s time. The 12 “Phases” of light schedule steps allow for flexibility in setting up complex lighting patterns.

**Figure 3:**
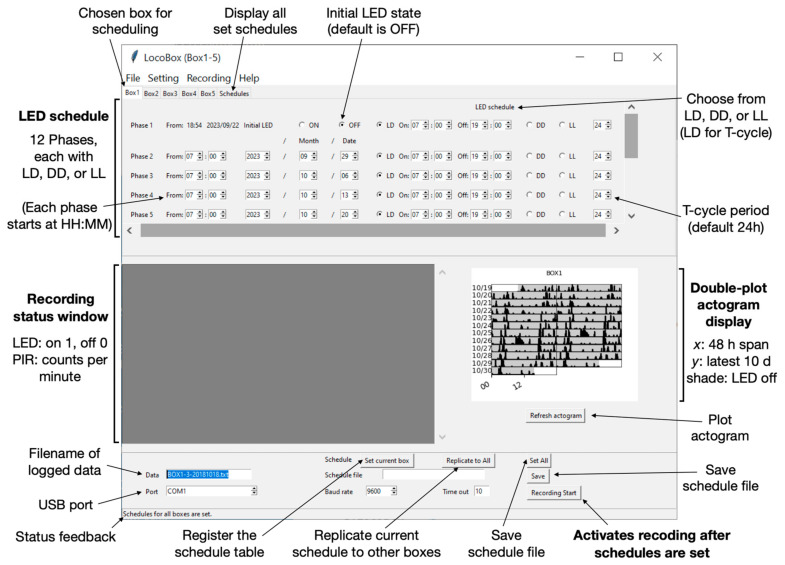
**LocoBox Python Interface**. The LocoBox interface can manage and record data from up to five LocoBoxes at once. A schedule set in one box can be duplicated to the others. Recording only initiates once all box schedules are configured. The process is started by pressing the “Recording Start” button. The double-plot actogram can be generated both online and offline; offline generation is accomplished by specifying the data file in the Filename window and pressing “Refresh actogram”.

The Arduino’s main loop interprets the schedule table to accurately handle scenarios, like jet lag or light–dark cycle inversions. The schedule table lists phases, each specifying just one lights-on and one lights-off time. This is crucial, especially when the table presents ambiguities, such as a light-off time at 1:00, which should be understood as 25:00, indicating a 6-h phase delay. This ambiguity is logically addressed by comparing the current LED state to the upcoming one using conditionals. For light–dark (LD) schedules that span more than 24 h, the system checks the current time against redefined ON and OFF times. If the ON time precedes the OFF time within the same day, the light stays ON during this interval and turns OFF afterward. If the ON time is after the OFF time, i.e., it extends into the next day, the light remains ON from the ON time through midnight and continues until the OFF time the next day. Ambiguities are further clarified by the initiation time, labeled “From:”, of a phase (Figure 3). For instance, when transitioning from constant darkness (DD) to constant light (LL), the light-on timing is determined by this initiation time (hour and minute specified). Since we have 12 phases, a complex lighting schedule is possible, independently and concurrently, across 5 boxes.

Automated T-cycle scheduling is enabled through LocoBox’s internal timekeeping, which can run independent phase cycles at arbitrarily defined period *T* (default 24 h, see Figure 3). Users can specify the *T* for each phase, and the Arduino updates its internal time with respect to the external time cycle (24 h) by the following formula, with both times referenced to Unix time:

(Internal time) += 24/*T* × (sampling duration in seconds) (1)

where += denotes in-place addition. The sampling duration (currently set at 60 s in version BTLocoBox_0003_v3) and initial timestamp are controlled by the RTC. The RTC ensures that that delays between iterations in the main loop do not influence the time precision.

Python was chosen as the programming language for the interface because of its popularity due to its open-source nature, clear and concise syntax, versatility across various applications, and robust community support. The pySerial package mediates serial communication between Python and Arduino, while the Tkinter package facilitates the GUI environment of the interface. The Python interface can organize complex LED scheduling through 12 sequential phases. In each phase of the “LED schedule”, when the LD radio button is selected, both the light-on and light-off times are set by specifying the date, hour, and minute. For constant dark or light conditions, the DD or LL radio buttons can be toggled, respectively. The interface can operate 5 LocoBoxes simultaneously, and the same schedule can propagate to other boxes by clicking “Replicate to All”. Schedules are registered by clicking “Set current box” or “Set All” for simultaneous registration across all 5 boxes. The “Recording Start” button becomes active only after the schedules are registered. Users can save the schedule file in the JSON format. “Baud rate” and “Time out” represent serial communication options, with default values of 9600 and 10, respectively. The “Port” specifies the USB connection, labeled as ‘tty’ in Linux and macOS, and ‘COM’ in Windows. The Port setting is designed to detect available USB ports, but this needs to be verified, especially when more than two LocoBox interfaces are run on the same computer. The Status bar indicates actions carried out by the software and returns warnings if errors occur (e.g., the user forgets to set the schedule, or the Arduino becomes disconnected from the computer). The Recording status sub-window displays the LED state (1 for on and 0 for off) and the currently recorded PIR counts. The file name for the logged data, saved in a tab-separated variable text format, should be entered in the “Data” field prior to recording. Upon pressing the “Recording Start” button, light scheduling and data logging commence (Figure 3). This continues until the LocoBox interface is closed.

The LocoBox is designed to support long-term recording. To prevent memory saturation, the stable version (BTLocoBox_0000_v0) of the interface omits online actogram analysis. In a later iteration, we have addressed this by using only data from the last 10 days instead of the entire dataset (BTLocoBox_0000_v2). In addition, the plotting function runs on a separate thread to ensure no interference with the on-going recording. This feature is handy to promptly visualize data, reducing the likelihood of errors in schedule settings and long-term recording malfunctions within LocoBox. Data, including LED status and PIR counts per minute, are logged and saved every minute for all 5 boxes. The first column logs the time (hour, minute, and second), while the second logs the date. From the third column onward, columns alternate between LED status and PIR counts for each box, continuing through the fifth box.

***Spectral Analysis of Locomotor Activities and Circadian Heatmap***: Time-dependent periods were calculated using a sliding window short-time Fourier transform (STFT) to create circadian heatmaps of power spectral density and phase angle, as previously described [19]. In brief, locomotor activity data with a one-minute resolution were first smoothed using a Hodrick–Prescott (HP) filter (penalty parameter λ = 5.184 × 10^7^) and detrended with the heavily HP filtered trend of the activity. From the smoothed and detrended data, spectral components were decomposed with STFT in a 5-day sliding window, moving one day at each time step. The final converted data were presented as spectrograms: power spectral density over a period range (reciprocal of frequency) for each time point, and phase angle over a 24-h period. These analyses were performed using Scipy’s signal processing toolbox (version 1.11.1) implemented in Python (version 3.11.5). We employed the spectral method because there were multiple embedded fundamental periods outside the 24-h period scale.

## 3. Results

**Figure 4:**
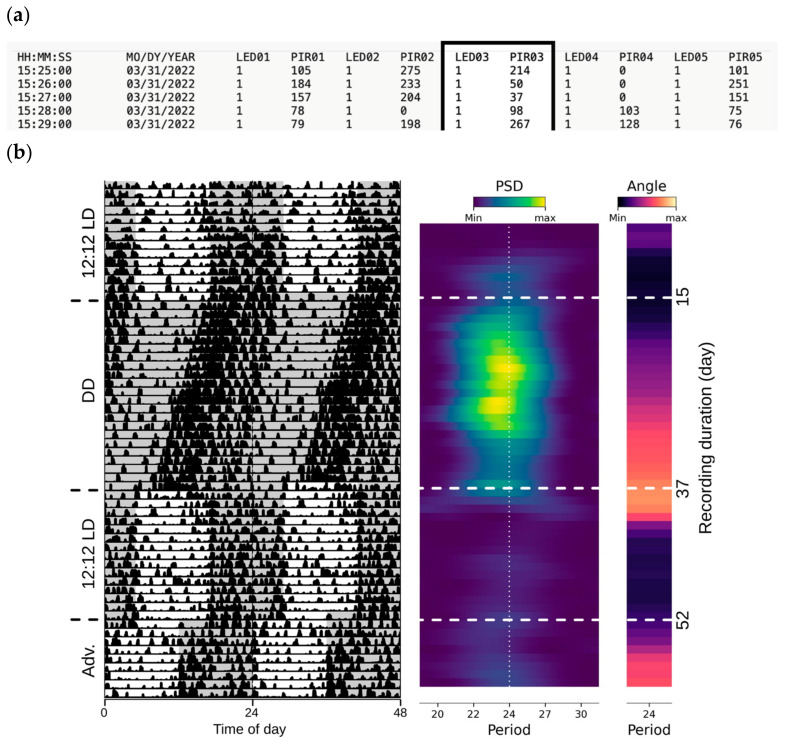
**Reading the data file and plotting the double-plot actogram**. (**a**) Presented is a partial data file excerpt acquired simultaneously from 5 LocoBoxes, each containing a cage with a single mouse. (**b**) Left: A double-plot actogram is generated using the PIR03 column, with the light–dark pattern (shaded regions indicate the dark period) derived from LED03 column (data from Box 3). Middle: Power spectral density (V^2^/86400 Hz) estimated from a sliding window of 5 days. Right: Phase angle (radian) estimated for the 24-h period.

Recording long-term locomotor activities is achievable with the LocoBox, without losing precision in timestamping. The data from all five boxes are acquired in less than 1 min, and the Arduino waits for the RTC seconds to reach exactly 00 before sending the timestamped data to the computer. We demonstrate a recording spanning approximately 2 months, captured using the LocoBox hardware and software (Figure 4). Data were recorded at 1-min intervals, with movement counts derived from the PIR. The datafile, saved in tab-separated variable (TSV) format, contains timestamps, LED state (1 for ON, 0 for OFF), and integrated locomotor activities for 1 min for all five boxes. This locomotor activity data can be represented in an actogram. A circadian actogram is a graphical representation that plots an organism’s activity level over a duration, often spanning several days or more. It is typically presented as a bar or filled line graph with time on the *x*-axis and activity level on the *y*-axis. To enhance the visualization of behavioral rhythms, actograms frequently use a double-plotted format where two consecutive days are aligned side by side, such as day 1 on the left and day 2 on the right [20]. This format enables easy detection of periodicity, which will appear as a vertically slanted straight line. In our double plot, activities from two consecutive days are depicted as miniature bar charts, with the LED state shown as background shades (white for light on and grey for light off), as previously described [19].

**Figure 5:**
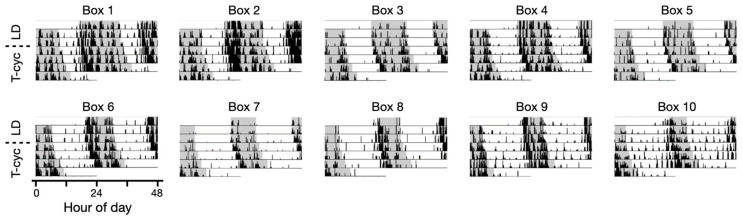
**Parallel entrainment of longer-than-24 h T-cycle combined with seasonal daylengths**. The versatility of LocoBox entrainment is demonstrated through simultaneous entrainment in 10 LocoBoxes with 25 h cycles under various day-lengths.

These recordings show that the circadian locomotor activities can be reliably recorded from the home cage as in the normal breeding environment, while the local light environment is controlled by the computer. The locomotor activities display the expected circadian trends, such as nocturnal behavior during the 12:12 light–dark (LD) cycles and a slightly-less-than 24-h periodicity under constant darkness (DD). This behavior is typical for the C57BL/6J strain. The phase delay resulting from transitioning from the prolonged DD to LD, as well as the phase advance following the 6-h jet lag light–dark condition, are well captured in this recording. The same trend is consistently observed across all 5 LocoBoxes. The LocoBox is capable of handling more complex entrainment scenarios, which can be performed in parallel in individual boxes. For example, T-cycles (non-24 h light–dark cycles) [20] can be combined with seasonal day-lengths to mimic the seasons of Mars (24.7-h cycle) at different latitudes (Figure 5).

## 4. Discussion

By combining Arduino and Python, the LocoBox creates an accessible system for circadian researchers, allowing them to explore a multitude of light–dark patterns without the need for manual interventions. The Arduino is a popular and affordable microcontroller, and Python provides an accessible interface for interaction with it. The software set is available on GitHub and has been continuously updated since 2018, allowing contributions from other users. The affordability and accessibility of this system hold potential for democratizing circadian research. It eliminates the need for expensive equipment by offering a customizable, yet standard, open-source solution, enabling even small-scale laboratories to manipulate and record behavioral circadian rhythms through light entrainment.

Despite the increasing need for exhaustive parameter searches and massive data acquisition in circadian behavior studies, not all laboratories can afford the necessary equipment for conducting such experiments. Furthermore, the tools provided by commercial companies are usually not flexible enough for non-conventional manipulations of the cycles. This lack of access causes a serious disparity in scientific research: a growing number of labs and researchers from developing countries are barred from state-of-the-art research in the field of biology due to limited funding [21].

In recent years, the scientific community has developed a new tendency towards modifiable open-source hardware and software to fulfill specific requirements for creative experiments. One of the most popular devices is Arduino, a microcontroller used to monitor and control experimental hardware. Arduino lacks a customizable GUI, and the experimental parameters cannot be modified without directly altering the code. There is much room to improve the interface and develop new ways to interact with Arduino. For example, by integrating Python and Arduino, a microwave-based motion detector has been implemented to monitor home cage activity in mice [22]. However, the data is stored on an SD card instead of being sent directly to the computer, causing a delay in analyzing and checking data. More importantly, the software does not allow automatic scheduling for different light/dark protocols, so they must be manually adjusted to demonstrate the validity of the software. Another low-cost system, using an A/D converter to measure the housekeeping activities (HKA) of animals and a MATLAB-based software, Circa Analysis, to facilitate post-processing and drawing actograms, enables different photoperiod scheduling [23]. Home cage-attachable portable actimetry devices have been developed, but they lack environmental light programming capability [24]. An economical solution for actimetry has been proposed for the open field, but this cannot be used in a breeding cage [25]. 

As we have briefly demonstrated in Figure 5, there are numerous entrainment options to be explored for behavioral circadian rhythms. Our combination of a small entrainable light box and Python-based GUI light scheduling has successfully enabled parallel experiments in 60 LocoBoxes at our laboratory. The importance of a system like this lies in the lengthy nature of circadian activity recording, which blurs the boundary between breeding and experimentation, and the long-term effects of entrainment on the circadian clock have not been fully characterized yet. Pittendrigh writes in his final memoir how he discovered experimental clues for morning (M) and evening (E) oscillators through pure accident [1]. A student who was running constant light entrainment in hamsters kept it for more than three months due to negligence. It was in these datasets that the splitting of M and E oscillators occurred. Although circadian rhythms research has matured and many different light–dark patterns have been applied, much room still remains for parameter space exploration. For example, Michael Gorman’s lab showed unusual bifurcation behaviors after two light–dark cycles embedded in 24 h [26,27], which can be easily explored in our LocoBox scheduling system. These findings underline the need for more exhaustive and long-term characterization of input–output patterns of behavioral circadian rhythms in breeding conditions. Our simple and economical system presents a practical solution to this challenge.

## Figures and Tables

**Table 1:** **List of required components**. The hardware components for the LocoBox are affordable and easily obtainable from common suppliers (e.g., Digi-Key). The part number is indicated in parentheses. Prices are in USD. Asterisks (*) indicate components that support 5 LocoBoxes. The software components are open-source and can be downloaded online. Standalone software for Windows is provided for users without Python.

	Main Components	Major Dependencies
**Hardware**	Plexiglas sheets (3 mm thick, matt black)	Laser cutting and assembly service
	DC brushless fan (HA60151V4-1000U-A99) $5.37	AC/DC 12 V* (RAC03-12SE/277/W) $22.58
	LED (CXA1304-0000-000C00B40E3) $1.24	Diffuser lens (PLJT29) $4.26
	Dry reed relay (103020014) $3.90	Heat sink (882-200AB) $5.30 AC/DC 9 V* (RAC04-09SC/W) $11.49 2N3904 NPN transistor (2N3904BU) $0.36
	PIR sensor (101020020) $8.70	
	Arduino Mega 2560* (A000067) $48.40	RTC DS3231* (Product ID: 3028) $13.95
		Grove Mega Shield* (103020027) $6.00
**Software**	Arduino IDE (version 1.8.7)	RTCLib (DS3231 RTC)
	Python (version 3.7.0)	Tkinter (installed with Python 3.7.0)
	or standalone program (Windows)	PySerial (version 3.4)

## Data Availability

All software described can be downloaded and further developed from GitHub at https://github.com/JihwanMyung/LocoBox. Other details will be available upon reasonable request.
